# Patients with Chronic Obstructive Pulmonary Disease Suffer from Worse Periodontal Health—Evidence from a Meta-Analysis

**DOI:** 10.3389/fphys.2018.00033

**Published:** 2018-01-25

**Authors:** Quan Shi, Bin Zhang, Helin Xing, Shuo Yang, Juan Xu, Hongchen Liu

**Affiliations:** Department of Stomatology, Chinese PLA General Hospital, Beijing, China

**Keywords:** chronic obstructive pulmonary disease, periodontitis, periodontal health status, risk factor, meta-analysis

## Abstract

**Background and Objective:** It is widely accepted that there is an association between chronic obstructive pulmonary disease (COPD) and periodontitis. However, whether the periodontal status of the COPD patients is worse than that of the non-COPD subjects is seldom assessed. The findings currently available are inconsistent, some even contradictory. Therefore, we performed this meta-analysis to compare the periodontal health status of COPD patients and non-COPD subjects.

**Methods:** PubMed and Embase were searched for all of the eligible studies which comparing the periodontal status between COPD patients and non-COPD subjects. The results of periodontal parameters in each study were extracted and the mean differences and 95% confidence intervals (CIs) for each parameter were calculated to determine their overall effects.

**Results:** In total, 14 studies involving 3348 COPD patients and 20612 non-COPD controls were included and 9 periodontal indexes were analyzed. The mean differences (95% CIs) between COPD and non-COPD subjects for probing depth, clinical attachment loss, level of alveolar bone loss, plaque index, oral hygiene index, bleeding index, bleeding on probing, gingival index, and remaining teeth were 0.261 (0.020–0.501), 0.480 (0.280–0.681), 0.127 (0.000–0.254), 0.226 (0.043–0.408), 0.802 (0.326–1.279), 0.241 (−0.106 to 0.588), 6.878 (5.489–8.266), 0.364 (0.036–0.692), and −3.726 (−5.120 to −2.331), respectively.

**Conclusion:** In summary, this meta-analysis demonstrates that the COPD patients suffer from worse periodontal health status, indicated by deeper periodontal pockets, high level of clinical attachment loss, worse oral hygiene, more inflammation and bleeding in the gingival tissue, and lower number of remaining teeth. Nevertheless, considering the limitations in our meta-analysis, more high-quality, and well-designed studies focusing on the periodontal health of the COPD patients are required to validate our conclusion.

## Introduction

As a chronic progressive disease, chronic obstructive pulmonary disease (COPD) is a syndrome of progressive airflow limitation caused by chronic inflammation of the airways and lung parenchyma (Barnes, 2000). COPD, one of the most common and costly respiratory diseases, is a major cause of morbidity and mortality worldwide. It is reported that COPD has affected 174.5 millions of global population and resulted in more than 3 million deaths every year (GBD 2016 Disease Injury Incidence Prevalence Collaborators, 2016; Rabe and Watz, 2017). Besides, the number of cases and deaths is projected to increase further, especially in the developing countries, which means COPD will remain a major health-care problem for decades to come (Rabe and Watz, 2017). Therefore, it is necessary to better understand the complex disease mechanisms and relevant risk factors related to COPD.

Periodontal disease (mainly gingivitis and periodontitis) is a common infectious disease affecting supporting structures of the teeth (Pihlstrom et al., 2005). If left untreated, periodontitis would result in erosion of the alveolar bone, and tooth loss. It is reported that periodontal diseases are highly prevalent and may affect up to 90% of the world population (Pihlstrom et al., 2005). More importantly, mounting evidence shows that periodontitis is associated with many systemic diseases, including cardiovascular disease (Shi et al., 2016), type 2 diabetes (Kudiyirickal and Pappachan, 2015), Alzheimer's disease (Cerajewska et al., 2015), stroke (Straka and Trapezanlidis, 2013), and osteoporosis (Penoni et al., 2017), etc. The association between periodontitis and COPD has aroused great interest of the clinical doctors and researchers, not only because both periodontitis and COPD are chronic and progressive conditions characterized by neutrophilic inflammation with subsequent proteolytic destruction of connective tissues, but also because they share common risk factors such as age and smoking (Usher and Stockley, 2013; Hobbins et al., 2017).

In recent years, many studies have shown that periodontitis is a significant and independent risk factor of COPD (Prasanna, 2011; Zeng et al., 2012; Usher and Stockley, 2013). In addition, it is widely accepted that oral pathogens and inflammatory cytokines from periodontal lesions can induce systemic inflammation, which may contribute to the pathogenesis of COPD (Terpenning, 2001; Usher and Stockley, 2013; Hobbins et al., 2017). However, two aspects are worth our attention: first, to evaluate the association between periodontitis and COPD, there is a great variation in how authors define periodontitis (Hobbins et al., 2017); second, whether the periodontal status of the COPD patients is worse than that of the non-COPD subjects is seldom assessed and the findings available are inconsistent, some even contradictory, based on previous studies (Yildirim et al., 2013; Terashima et al., 2016). Moreover, despite the existence of two systematic reviews and meta-analyses evaluating the association between periodontitis and COPD (Scannapieco et al., 2003; Zeng et al., 2012), there are still a lack of specialized meta-analyses that quantitatively compare the periodontal status between the COPD and non-COPD subjects by evaluating the related clinical periodontal indexes.

To facilitate the determination of uniform statistical indicators and obtain more accurate results, it is preferable to collect and merge data from the same periodontal status index. Therefore, we performed this meta-analysis to identify all related studies comparing the periodontal status indexes between COPD patients and non-COPD subjects, and then evaluate whether the periodontal status of COPD patients is worse than that of the non-COPD subjects by comparing the clinical periodontal parameters (i.e., level of alveolar bone loss, bleeding index, bleeding on probing, etc.). We hope these results would facilitate a better understanding of the association between COPD and periodontal health, and provide clinicians and patients with better evidence-based evaluations and recommendations.

## Methods

### Search strategy and selection criteria

For this meta-analysis, we searched PubMed and Embase on August 16th, 2017 to identify related studies. The combination of the following key words was used: (“periodontitis” OR “periodontal disease”) AND (“COPD” OR “chronic obstructive pulmonary disease”), and the language was restricted to English. In addition, we also searched the reference lists of related papers identified by our searches for additional references.

To minimize selection bias, the inclusion and exclusion criteria were established before our search of the database. Eligibility criteria for this meta-analysis were: (1) study comparing the periodontal status between the COPD patients and non-COPD subjects; (2) the COPD patients were described clearly; (3) available data of periodontal indexes can be extracted; (4) the studies should use validated methods to perform periodontal examinations. Articles were excluded if: (1) review or case report; (2) *in vitro* or animal study; (3) the study lacks available data; (4) the periodontal parameters were affected by other interventions; (5) the case group in the study consisted of patients with other lung diseases.

Based on the above selection criteria, two reviewers (SQ and ZB) screened all of the studies independently. First, the reviewers screened all titles and abstracts independently to exclude the duplicate or obviously irrelevant articles. Then, the reviewers assessed the full-text of the remaining articles for inclusion. Finally, only the articles meeting the inclusion criteria were included in our meta-analysis. Any discrepancies were resolved by discussion between the reviewers.

### Data extraction and quality assessment

Two reviewers (SQ and ZB) used a standardized data extraction form to independently collect basic data from each included study. The information mainly included: name of the first author, publication year, study country, study design type, description of the study objects (sample size, sex and age of the COPD and non-COPD), periodontal parameters and the results (i.e., level of alveolar bone loss (ABL), bleeding index (BI), bleeding on probing (BOP), clinical attachment loss (CAL), gingival index (GI), probing depth (PD), plaque index (PI), oral hygiene index (OHI), number of remaining teeth).

In this meta-analysis, we used the Newcastle-Ottawa Scale (NOS) to assess the quality of the included studies. By this assessment tool, the methods of study selection, comparability of the study subjects, and outcome of exposure were evaluated to appraise the methodological quality of the included studies Wells et al. The NOS score ranges from 0 to 9. NOS scores of 0 to 3, 4 to 6, and 7 to 9 indicated low, moderate, and high study quality, respectively. All included studies were assessed by two authors (XH and YS) independently.

### Statistical analysis

The data analyses were performed by using STATA software (Version 12.0; Stata Corp, College Station, Texas, USA). For continuous data, weighted mean difference (WMD) with 95% confidence intervals (CIs) was calculated by pooling of the mean and standard deviations (*SD*) of each study. If dichotomous data available, odds ratios (OR) with 95% CI were calculated. *P*-value < 0.05 was considered statistically significant. Statistical heterogeneity between studies was tested using I^2^ statistics. A fixed effects model was used if the I^2^ < 25%. I^2^ > 25% was considered to be substantial heterogeneity and the random effects was used.

Sensitivity analysis was made by removing one study each time in order to analyze the stability of the pooled results, if there were enough studies in the comparison (≥5 studies). The publication bias was detected by the Egger's test if there were more than 10 articles in the comparison of each periodontal index. The agreement between two authors were determined by the Kappa statistic.

## Results

### Summary of the included studies

**Figure 1:**
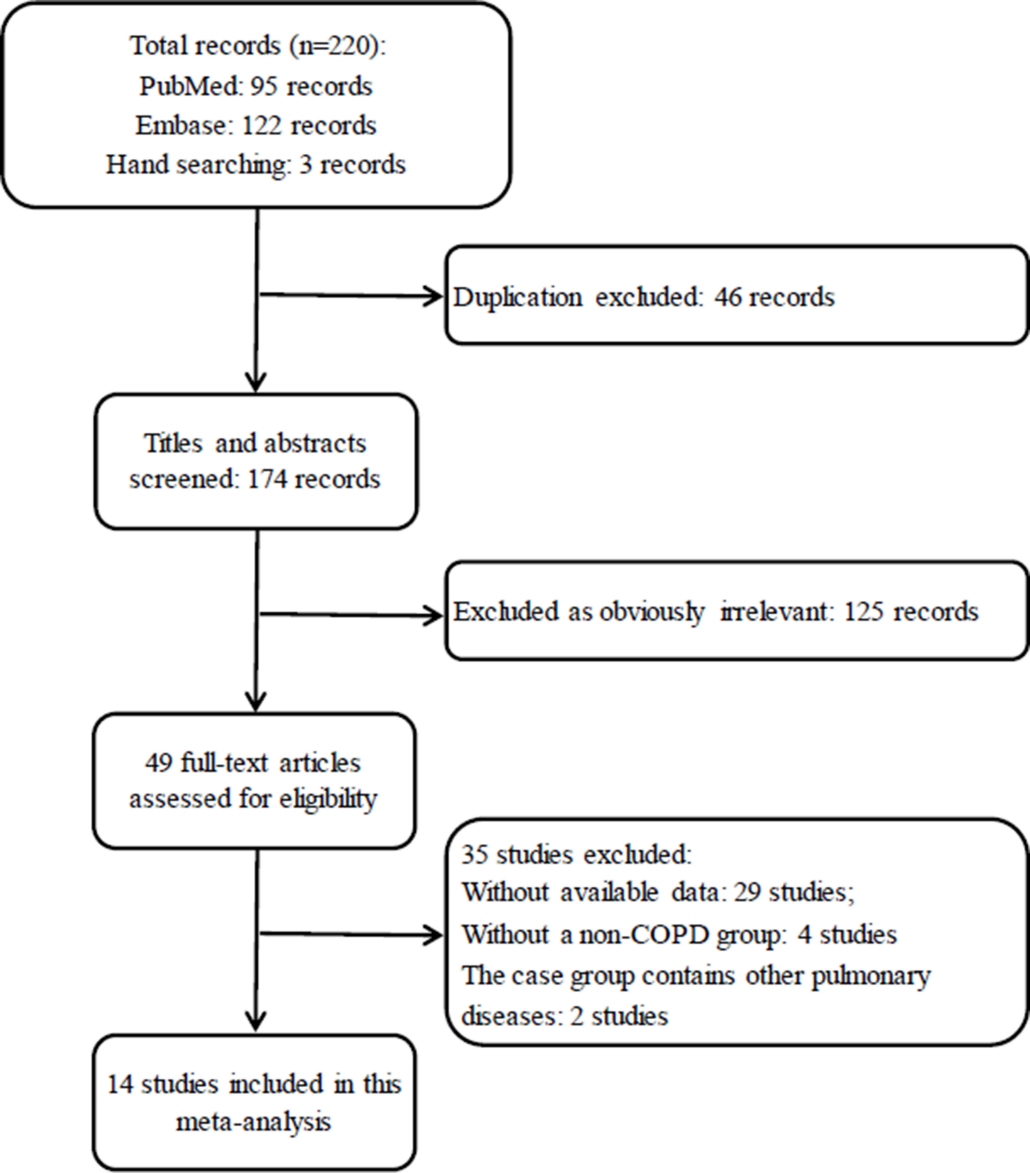
Flow diagram for the selection of studies. COPD, chronic obstructive pulmonary disease.

In total, 220 records were identified after an initial search based on our previous research strategies. And 171 records which are irrelevant or duplicate were excluded first after the titles and abstracts were screened, leaving 49 articles for a further full-text review. Eventually, 35 studies were excluded and 14 studies (Hayes et al., 1998; Scannapieco and Ho, 2001; Deo et al., 2009; Wang et al., 2009; Prasanna, 2011; Si et al., 2012; Ledić et al., 2013; Peter et al., 2013; Vadiraj et al., 2013; Yildirim et al., 2013; Öztekin et al., 2014; Bhavsar et al., 2015; Chung et al., 2016; Terashima et al., 2016) were eligible for inclusion in this analysis to perform the comparison of periodontal status between COPD and non-COPD subjects. The selection procedure is shown in Figure 1.

The publication dates of the 14 included studies ranged from 1998 to 2016. Among the 14 studies, 12 (Hayes et al., 1998; Deo et al., 2009; Wang et al., 2009; Prasanna, 2011; Si et al., 2012; Ledić et al., 2013; Peter et al., 2013; Vadiraj et al., 2013; Yildirim et al., 2013; Öztekin et al., 2014; Bhavsar et al., 2015; Terashima et al., 2016) are case-control study (one of which is nested case-control study) and 2 are cross-sectional studies (Scannapieco and Ho, 2001; Chung et al., 2016). Totally 3348 COPD patients and 20612 non-COPD controls were studied. Characteristics of included studies and patients are shown in Table 1.

**Table 1:** Characteristics of included studies.

**Study ID (First author, Year)**	**Nation**	**Design**	**Case (COPD)**		**Control (non-COPD)**		**Periodontal indexes**	**NOS score**
			**Number (male/female)**	**Age (mean ±*SD*)**	**Number (male/female)**	**Age (mean ±*SD*)**		
Terashima T, 2016	Japan	C-C	60 (55/5)	73.9 ± 7.6	76 (49/27)	65.1 ± 5.8	Remaining teeth, PD	6
Chung JH, 2016	Korea	C-S	697 (524/173)	64.3 ± 0.2	5181 (2086/3095)	54.6 ± 0.1	Remaining teeth	6
Bhavsar NV, 2015	India	C-C	100 (62/38)	25–75^a^	100 (57/43)	25–75^a^	PI, GI, OHI, PD, CAL	5
Öztekin G, 2014	Turkey	C-C	52 (49/3)	57.5 ± 9.7	38 (32/6)	53.5 ± 9.2	Remaining teeth, PI, GI, BOP, PD, CAL	7
Peter KP, 2013	India	C-C	102 (92/10)	59.48 ± 11.13	399 (303/96)	49.69 ± 10.16	CAL, PD, OHI, PI, GI	5
Ledić K, 2013	Croatia	C-C	93 (65/28)	65.75 ± 9.65	43 (18/25)	62.12 ± 11.91	Remaining teeth, BI, PD, CAL	5
Vadiraj S, 2013	India	C-C	50 (34/16)	48.9 ± 12.1	50 (32/18)	44 ± 9.9	CAL	5
Yildirim E, 2012	Turkey	C-C	36 (34/2)	68.9 ± 8.8	20 (19/1)	59.5 ± 9.1	PD, CAL, BOP	7
Si Y, 2012	China	C-C	581 (422/159)	63.9 ± 9.4	438 (218/220)	62.8 ± 9.5	Remaining teeth, PD, BI, PI, CAL, ABL	6
Prasanna, SJ 2011	India	C-C	50 (50/0)	56.3 ± 3.8	50 (27/23)	47.4 ± 4.9	GI, BI	4
Deo V, 2009	India	C-C	150 (140/10)	41.43 ± 7.53	50 (38/12)	43.62 ± 5.53	OHI, CAL	5
Wang Z, 2009	China	C-C	306 (210/96)	63.94 ± 9.84	328 (164/164)	63.26 ± 8.98	Remaining teeth, PD, CAL, BI, PI, ABL	7
Scannapieco FA, 2001	USA	C-S	810 (506/304)	51.2 ± 17.9	12982 (6821/6161)	43.9 ± 17.7	CAL	5
Hayes C, 1998	USA	NCC	261 (261/0)	45.05 ± 9.7	857 (857/0)	42.18 ± 9.1	Remaining teeth	6

a *The range of subjects' age. COPD, chronic obstructive pulmonary disease; C-C, case-control study; C-S, cross-sectional study; NCC, nested case-control study; SD, standard deviation; NOS, Newcastle-Ottawa Scale; ABL, level of alveolar bone loss; BI, bleeding index; BOP, bleeding on probing; CAL, clinical attachment loss; GI, gingival index; PD, probing depth; PI, plaque index; OHI, oral hygiene index*.

Methodological quality assessments of the included studies are also shown in Table 1. Among the 14 studies, 3 studies (Wang et al., 2009; Yildirim et al., 2013; Öztekin et al., 2014) scoring 7 points were considered to be of high quality and 11 studies (Hayes et al., 1998; Scannapieco and Ho, 2001; Deo et al., 2009; Prasanna, 2011; Si et al., 2012; Ledić et al., 2013; Peter et al., 2013; Vadiraj et al., 2013; Bhavsar et al., 2015; Chung et al., 2016; Terashima et al., 2016) were considered to be of moderate quality because their score ranged from 4 to 6 points. While the Kappa value for the article screening and quality assessment were 0.892 and 0.811, respectively, which mean an excellent agreement between the authors.

### Meta-analysis of the periodontal status between COPD and non-COPD

#### Probing depth

**Figure 2:**
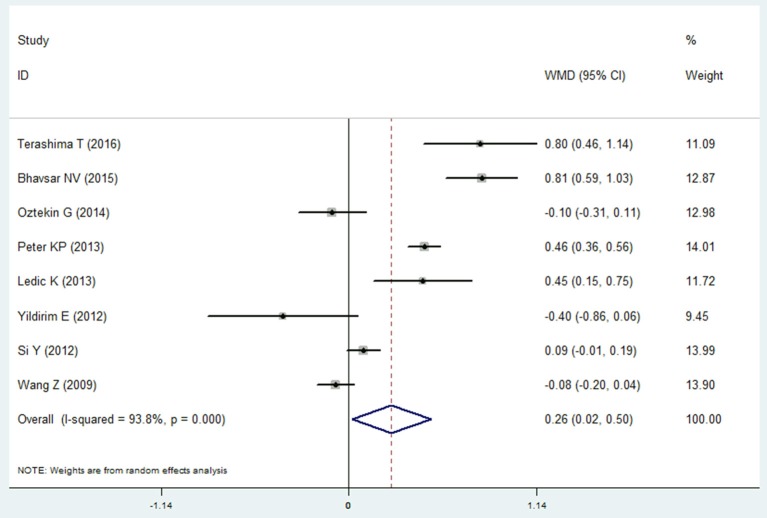
Forest plot of the mean difference in probing depth between the COPD and non-COPD groups.

Eight studies reported the data of probing depth in the COPD and non-COPD group (Wang et al., 2009; Si et al., 2012; Ledić et al., 2013; Peter et al., 2013; Yildirim et al., 2013; Öztekin et al., 2014; Bhavsar et al., 2015; Terashima et al., 2016). The results of meta-analysis revealed that **probing depth** in the COPD patients was deeper than that of the non-COPD group, and the mean difference was 0.261 mm (95% CI: 0.020–0.501, *P* = 0.033). Because the heterogeneity between the studies was high (I^2^ = 93.8%), a random effects model was selected (Figure 2, Table 2). However, the sensitivity analysis revealed that the results were not stable. When remove the Terashima T 2016, Bhavsar NV2015, Peter KP 2013, or Ledić K 2013, the pooled results were of no significance (Table S1).

**Table 2:** Summary of the periodontal indexes analyzed in this meta-analysis.

**Index**	**Number of studies**	**WMD**	**95%CI**	***P***	**I^2^ (%)**
Probing depth	8	0.261	0.020–0.501	0.033	93.8
Clinical attachment loss	10	0.480	0.280–0.681	0.000	89.7
Level of alveolar bone loss	2	0.127	0.000–0.254	0.050	88.1
Plaque index	5	0.226	0.043–0.408	0.016	95.8
Oral hygiene index	3	0.802	0.326–1.279	0.001	94.9
Bleeding index	4	0.241	−0.106 to 0.588	0.173	95.9
Bleeding on probing	2	6.878	5.489–8.266	0.000	0.0
Gingival index	4	0.364	0.036–0.692	0.030	97.7
Remaining teeth	7	−3.726	−5.120–2.331	0.000	94.5

*WMD, weighted mean difference; CI, confidence intervals*.

#### Clinical attachment loss

**Figure 3:**
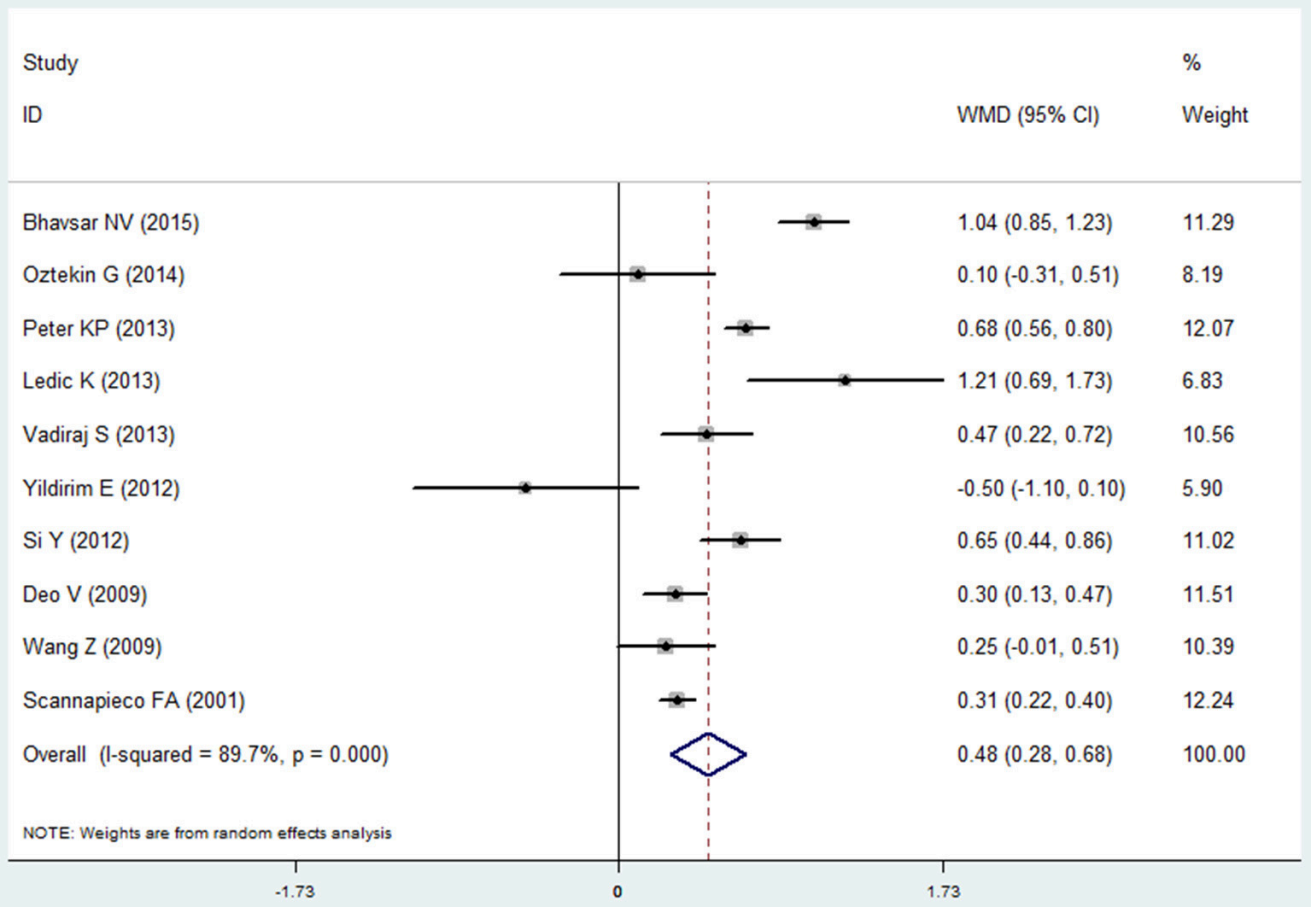
Forest plot of the mean difference in clinical attachment loss between the COPD and non-COPD groups.

Ten studies reported the results of clinical attachment loss in the COPD and non-COPD group (Scannapieco and Ho, 2001; Deo et al., 2009; Wang et al., 2009; Si et al., 2012; Ledić et al., 2013; Peter et al., 2013; Vadiraj et al., 2013; Yildirim et al., 2013; Öztekin et al., 2014; Bhavsar et al., 2015). The overall effects of the meta-analysis revealed that the COPD patients suffered more clinical attachment loss compared with the non-COPD subjects. The mean difference was 0.480 mm (95% CI: 0.280–0.681, random effect model, I^2^ = 89.7%), which was of statistical significance (*P* = 0.000, Figure 3, Table 2). The Egger's test revealed that no publication bias was detected (*P* = 0.979). Besides, the sensitivity analysis revealed that the pooed results were robust (Table S1).

#### Level of alveolar bone loss

**Figure 4:**
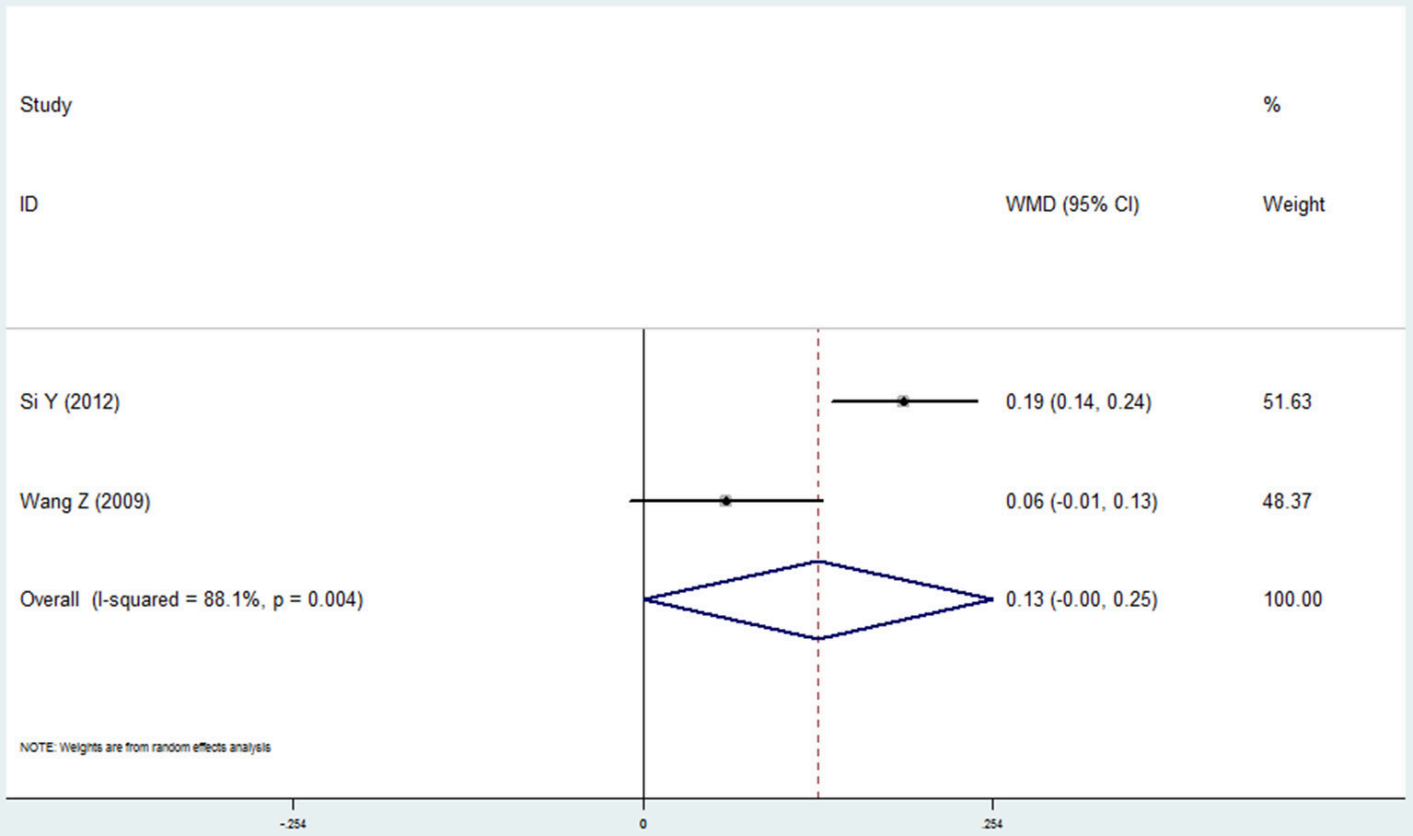
Forest plot of the mean difference in level of alveolar bone loss between the COPD and non-COPD groups.

Two studies reported the results of level of alveolar bone loss assessment in the COPD and non-COPD group (Wang et al., 2009; Si et al., 2012). A borderline level of statistical significance was found in this assessment. The mean difference was 0.127 (95% CI: 0.000–0.254, *P* = 0.050, random effect model, I^2^ = 88.1%). The results are shown in Figure 4, Table 2.

#### Plaque index

**Figure 5:**
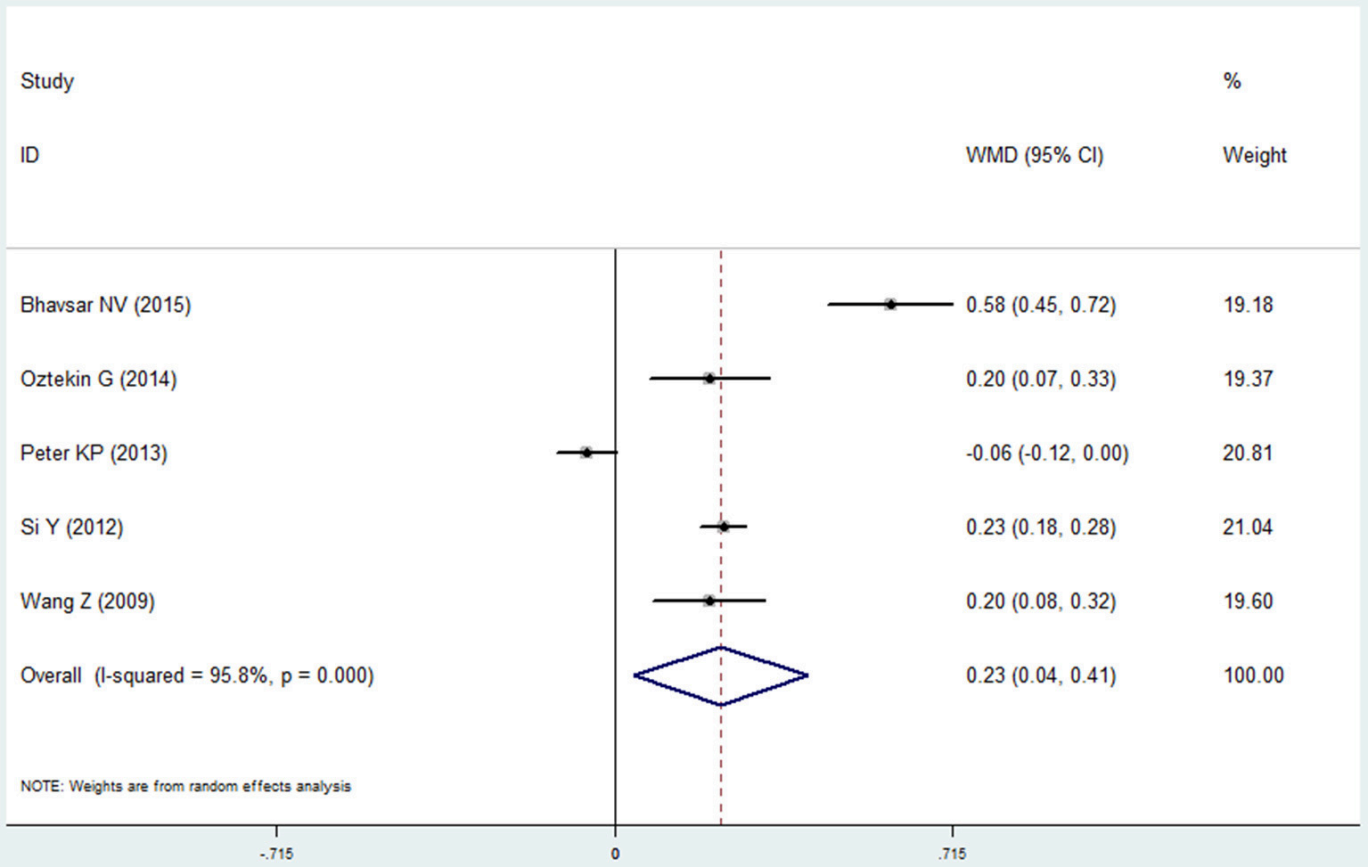
Forest plot of the mean difference in plaque index between the COPD and non-COPD groups.

Five studies reported the results of plaque index in the COPD and non-COPD group (Wang et al., 2009; Si et al., 2012; Peter et al., 2013; Öztekin et al., 2014; Bhavsar et al., 2015). More dental plaque and soft matter were observed in the COPD group, indicated by the higher score of this group (mean difference=0.226, 95% CI: 0.043–0.408, *P* = 0.016, random effect model, I^2^ = 95.8%, Figure 5, Table 2). While as shown in the Table S1, the pooled results were not stable when performing the sensitivity analysis was made by removing one study each time.

#### Oral hygiene index

**Figure 6:**
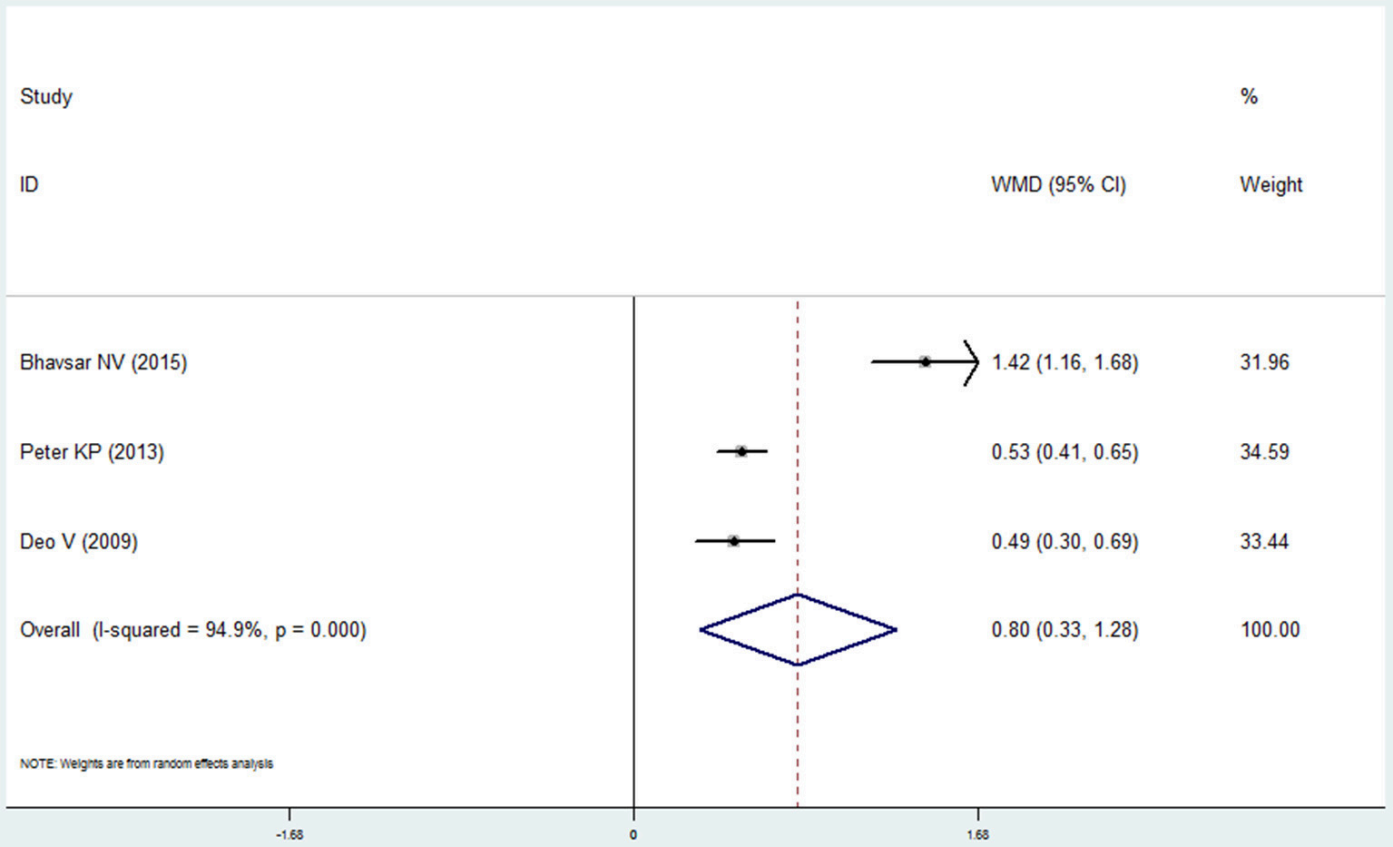
Forest plot of the mean difference in oral hygiene index between the COPD and non-COPD groups.

Three studies reported the data of the oral hygiene index in the COPD and non-COPD group (Deo et al., 2009; Peter et al., 2013; Bhavsar et al., 2015). Based our meta-analysis, the oral hygiene index score in the COPD patients was higher than that of the non-COPD subjects, which was of statistical significance (mean difference = 0.802, 95% CI: 0.326–1.279, *P* = 0.001, random effect model, I^2^ = 94.9%, Figure 6, Table 2).

#### Bleeding index and bleeding on probing

**Figure 7:**
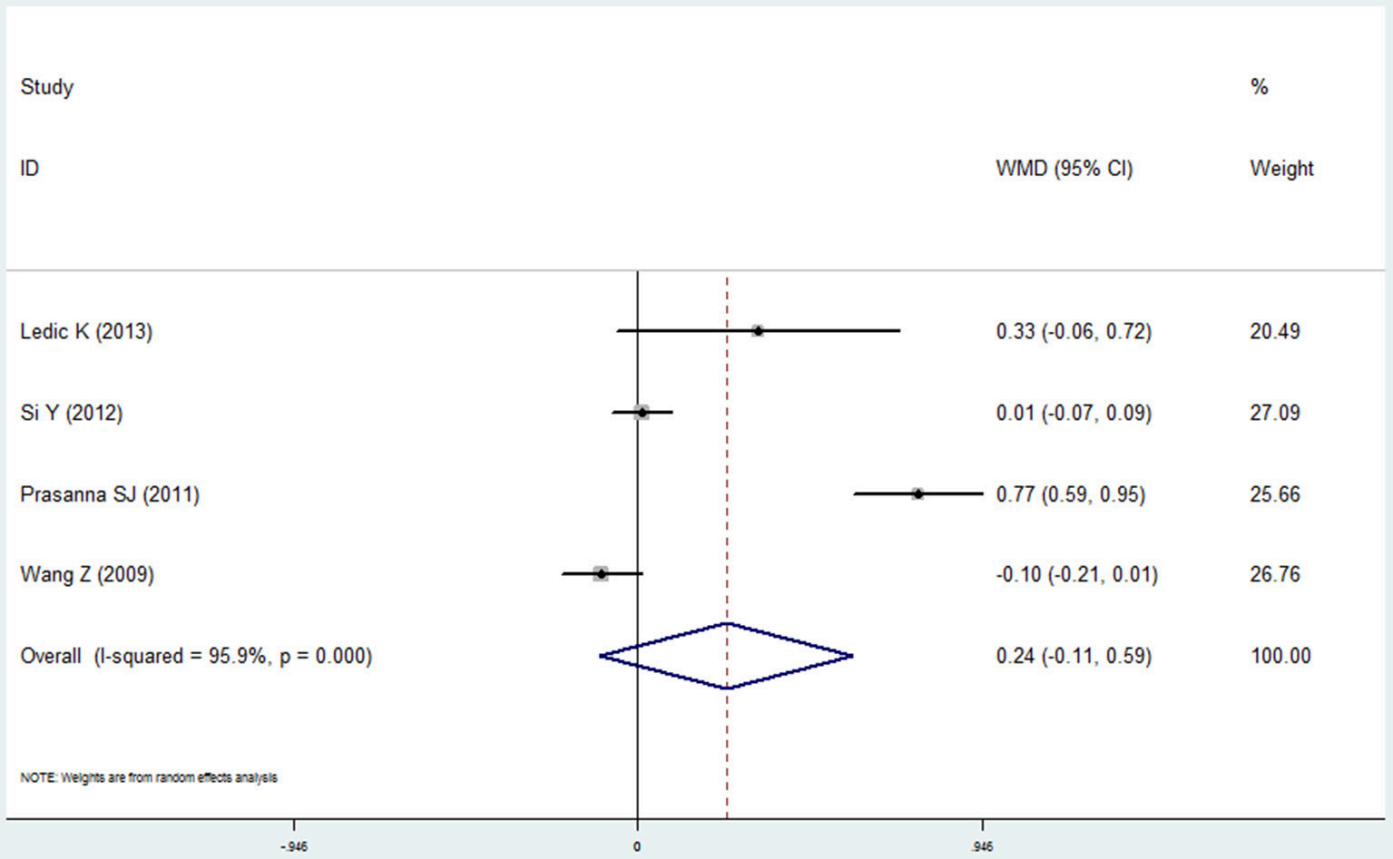
Forest plot of the mean difference in bleeding index between the COPD and non-COPD groups.

**Figure 8:**
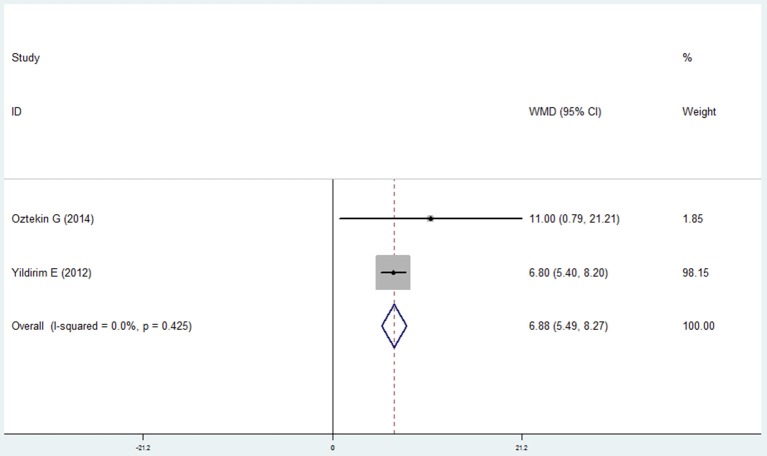
Forest plot of the mean difference in bleeding on probing between the COPD and non-COPD groups.

Four studies (Wang et al., 2009; Prasanna, 2011; Si et al., 2012; Ledić et al., 2013) reported the results of the bleeding index and 2 studies (Yildirim et al., 2013; Öztekin et al., 2014) reported the bleeding on probing in the COPD and non-COPD group. However, contrary results were observed by comprehensive analysis. For the bleeding index, the mean difference was 0.241 with no statistical significance (95% CI: −0.106 to 0.588, *P* = 0.173, random effect model, I^2^ = 95.9%, Figure 7), while the results of bleeding on probing revealed that more bleeding was observed in the COPD (mean difference = 6.878, 95% CI: 5.489–8.266, *P* = 0.000, fixed effect model, I^2^ = 0%, Figure 8, Table 2).

#### Gingival index

**Figure 9:**
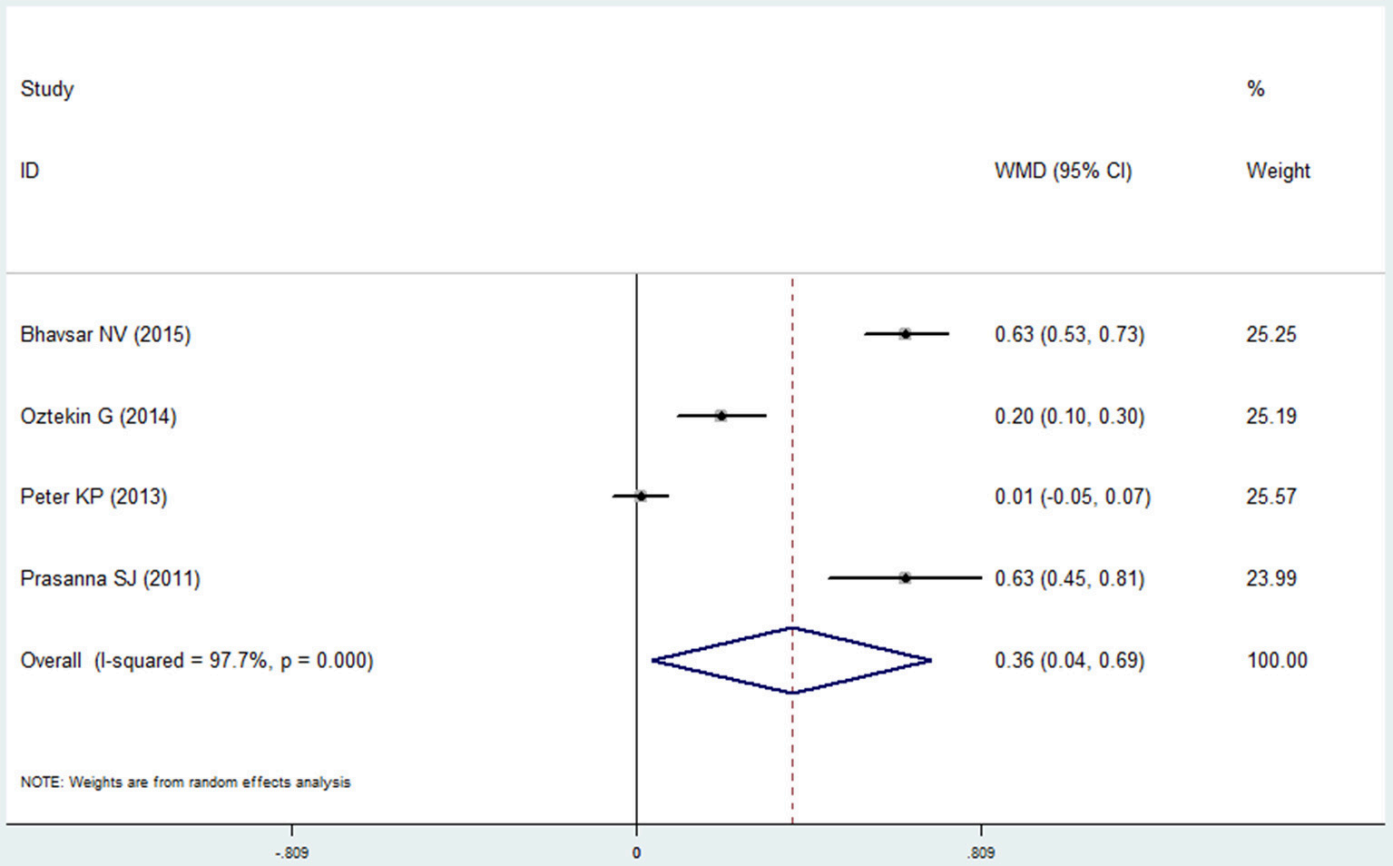
Forest plot of the mean difference in gingival index between the COPD and non-COPD groups.

Four studies reported the data of gingival index in the COPD and non-COPD group (Prasanna, 2011; Peter et al., 2013; Öztekin et al., 2014; Bhavsar et al., 2015). The condition of the gingiva in the COPD group was worse than that in the non-COPD group, and the mean difference between the groups was 0.364 after the pooled analysis, which showed statistical significance (95% CI: 0.036–0.692, *P* = 0.030, random effect model, I^2^ = 97.7%, Figure 9, Table 2).

#### Remaining teeth

**Figure 10:**
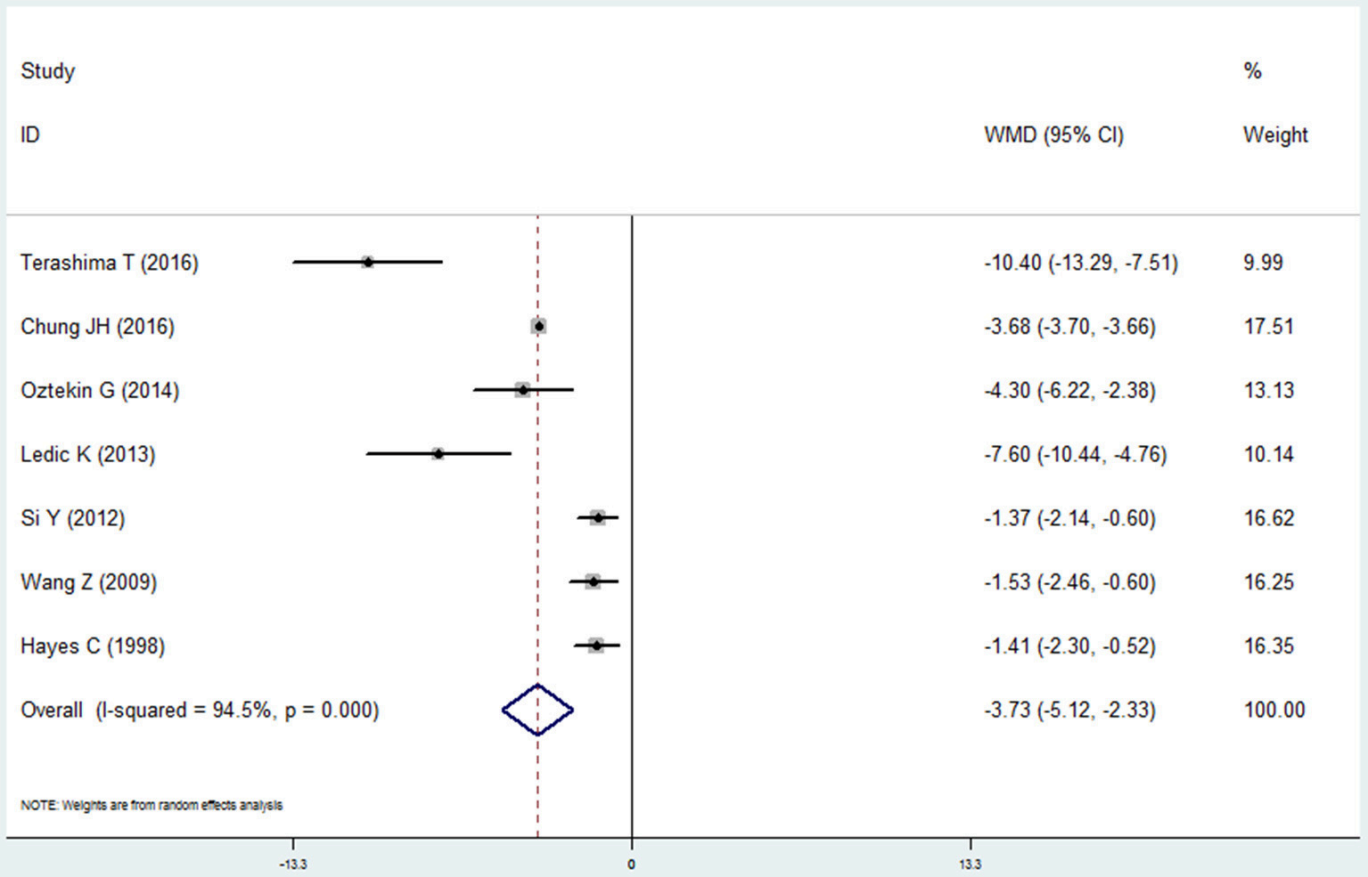
Forest plot of the mean difference in remaining teeth between the COPD and non-COPD groups.

Seven studies made the analysis of the remaining teeth in the COPD patients and non-COPD subjects (Hayes et al., 1998; Wang et al., 2009; Si et al., 2012; Ledić et al., 2013; Öztekin et al., 2014; Chung et al., 2016; Terashima et al., 2016). The results of this meta-analysis revealed that the COPD patients had less remaining teeth than the non-COPD subjects. The mean difference was −3.726 (95% CI: −5.120 to −2.331, random effect model, I^2^ = 94.5%), which was statistically significant (*P* = 0.000, Figure 10, Table 2). The sensitivity analysis revealed that the results are stable (Table S1).

## Discussion

COPD and periodontitis are both chronic, progressive inflammatory conditions characterized by neutrophilic-mediated tissue damage (Sinden and Stockley, 2010; Scott and Krauss, 2012; Hobbins et al., 2017). Based on the previous studies, we hypothesized that compared with the non-COPD subjects, the COPD patients may suffer from worse periodontal health status. However, there are studies reporting mixed results in the comparison of the periodontal status. Some of the findings are even contrary (Yildirim et al., 2013; Terashima et al., 2016). Therefore, we performed this meta-analysis to explore the periodontal health status between the COPD patients and non-COPD subjects.

Totally, 14 studies were included in our meta-analysis, and 3348 COPD patients and 20612 non-COPD controls were studied. Nine periodontal health indexes were extracted from the included studies and pooled to compare the periodontal status between the COPD patients and non-COPD subjects. Among these 9 indexes, the results of 7 indexes confirmed worse periodontal health, worse oral hygiene and less remaining teeth in the COPD patients. The results of level of alveolar bone loss revealed a borderline level of statistical significance between the two groups (*P* = 0.05). As for the bleeding index, despite the mean difference revealed more bleeding in the COPD, the results did not show statistical difference (*P* = 0.173). These results provide some support for our original hypothesis.

All of the 9 periodontal indexes are common and important parameters to evaluate periodontal status and the severity of the periodontal disease in both clinical research and clinical practice. Probing depth, clinical attachment loss and level of alveolar bone loss are the most commonly used variables to evaluate the loss of the soft (periodontal ligament) and hard (alveolar bone) tissue (Machtei et al., 1993; Kingman and Albandar, 2002; Page and Eke, 2007; Leroy et al., 2010). In our meta-analysis, COPD patients had deeper periodontal pockets and more clinical attachment loss, which means periodontitis condition in the COPD group is more severe than that in the non-COPD group. Oral hygiene is a basic factor for oral health. Poor oral hygiene can cause gingivitis and periodontitis, eventually leading to tooth loss (Bakdash, 1994). In this meta-analysis, plaque index and oral hygiene index were analyzed to compare the oral hygiene between the COPD and non-COPD group. The results indicated a significantly higher level of dental plaque and worse oral hygiene in the COPD patients. In addition, the inflammation and bleeding of the gingival tissue was evaluated by the gingival index, bleeding index and bleeding on probing (Page and Eke, 2007). However, despite the results of these three indexes revealed that the high level of inflammation and more bleeding of the COPD patients, the results of gingival index and bleeding index are statistically significant, while the results of bleeding on probing is of no statistically significance. We think these different results may be caused by the limited number of studies in these three indexes, which means more well-designed studies are needed in the future. At last the number of remaining teeth in the two groups was evaluated and the overall effect revealed that COPD patients had less remaining teeth in the mouth than the non-COPD patients.

The common pathophysiological processes may play a critical role in these results. Compared with the non-COPD subjects, the COPD patients showed higher levels of circulating inflammatory cytokines and destructive mediators including C-reactive protein (CRP), interleukin (IL)-8, tumor necrosis factor (TNF) α, and matrix metalloproteinase (MMP) (Barnes and Celli, 2009; Sinden and Stockley, 2010). Similarly, the periodontitis have same pathophysiological processes and these same inflammatory cytokines and destructive mediators were also increased in the periodontitis patients, causing the loss of the dental ligamentous support and alveolar bone (Usher and Stockley, 2013; Hobbins et al., 2017). Besides, in the COPD patients, it is suggested that neutrophils are dysfunctional (Hoenderdos and Condliffe, 2013). These impaired neutrophil functions may make the host less able to deal efficiently with bacteria in the periodontium, cause the dysfunctional host-immune-inflammatory reaction in the periodontal tissues and increase the local inflammation (Meyle and Chapple, 2015). Moreover, the COPD and periodontitis share common risk factors, especially smoking (Hyman and Reid, 2004), which could also induce the damage of the periodontal tissues. Moreover, the medications used to treat COPD may contribute to the worse periodontal health of the COPD patients. Glucocorticoid, salbutamol, and tiotropium bromide are commonly used drugs in clinical situations. However, these drugs may have side effects on the other systems, including oral health. For example, glucocorticoid may inhibit bone formation, suppression of calcium absorption, leading to osteoporosis and loss of the alveolar bone (Bouvard et al., 2013; Sousa et al., 2017). While the salbutamol and tiotropium bromide may cause the reduction of salivary flow and dry mouth, which is bad for the teeth and periodontal health (Ryberg and Johansson, 1995; Keating, 2012).

In our meta-analysis, the comprehensive analysis performed within each index revealed high levels of heterogeneity except for the bleeding on probing, therefore, a random effect model was adopted in the comparison of the other eight indexes. Besides, the sensitivity analysis revealed that the results of the clinical attachment loss and remaining teeth are stable, while not in the probing depth and plaque index. In our opinion, three factors may contribute to the high heterogeneity and the unstable pooled results. First, the design of the included studies is different, 12 of which are case-control studies and the other two are cross-sectional studies. Second, different studies match different confounding factors between the COPD group and non-CODP group, especially for the common factors shared by the periodontitis and COPD, such as age, smoking, and socio-economic status. Three, the number of the studies included in the currently analysis is limited, and in which only three studies are of high quality. It is well-known that the more the high quality studies included, the more reliable results can we get. Because only observational studies were included in our meta-analysis, representativeness of the cases and controls, comparability of cases and controls and the structured interview where blind to case/control status are all very important to help us to get reliable and accurate conclusions. Moreover, all of these aspects determined the quality of the included studies. Therefore, well-designed high quality studies are needed in the future.

In a large scale cohort study, Shen et al. found that the overall incidence of periodontal diseases was 1.19-fold greater in the COPD group than in the comparison group, which means that patients with COPD are at a higher risk of developing periodontal diseases than the general population (Shen et al., 2015). This result provides new evidence to determine the causal relationship between COPD and periodontitis. It is consistent with our meta-analysis. Namely, the periodontal health of the COPD patients is worse than that of the control subjects. Besides, Shen et al. also performed a retrospective cohort study to evaluate whether the treatment of periodontal diseases was associated with beneficial outcomes for COPD patients. After a 5-year follow-up, they found that the incidence rates of adverse respiratory events were significantly lower in the treatment group than in the comparison group (Shen et al., 2016). Hence they found that adequate periodontal health care is important for COPD patients with periodontal diseases. Moreover, Kucukcoskun et al. conducted a prospective, controlled group trial to assess the effect of initial periodontal therapy on exacerbation frequency in COPD patients (Kucukcoskun et al., 2013). The test group showed a significant reduction in the exacerbation frequency (from 3 to 2) during the 12-month follow-up, while the control group increased from 2 to 3 (Kucukcoskun et al., 2013). These results indicated that we should pay more attention to the periodontal health of the COPD patients. Active treatment of the periodontal disease may have more beneficial effects on the COPD patients, but more studies are needed to confirm it.

To our knowledge, this is the first meta-analysis to compare the periodontal status between the COPD patients and non-COPD subjects, which provides more information for understanding the relationship between the COPD and periodontal disease. However, there are three limitations we must point out. First, although 14 studies are included, the number of studies in each index is limited, especially for the level of alveolar bone loss, oral hygiene index and bleeding on probing. Besides, only three studies are of high quality and the others are of moderate quality. Second, because of the limited number of the studies, no subgroup analysis was performed, and publication bias was only performed in the analysis of clinical attachment loss in this meta-analysis. Third, because of the limited information provided by the included studies, we are not able to explore the effects of the common risk factors of the two diseases on the overall effects.

In summary, this meta-analysis supports the hypothesis that compared with the non-COPD subjects, the COPD patients suffer from worse periodontal health status, indicated by deeper periodontal pockets, high level of clinical attachment loss, worse oral hygiene, more inflammation and bleeding in the gingival tissue, and lower number of remaining teeth. In the clinical work, it is necessary to attach importance to the CODP patient's periodontal health. Considering the limitations of the current study, more high-quality and well-designed studies on the periodontal health of the COPD patients are required to validate our conclusion. Moreover, exploring the effect and mechanism of the treatment of periodontal disease on the COPD is worthy of further efforts.

## Author contributions

QS and BZ: literature research, study selection and data extraction; HX and SY: risk of bias evaluation and data analysis; QS, BZ, and JX: manuscript draft and revised; HL and JX are the corresponding authors, and they undertook the work of designing this meta-analysis. All authors read and approved the final manuscript.

### Conflict of interest statement

The authors declare that the research was conducted in the absence of any commercial or financial relationships that could be construed as a potential conflict of interest. The reviewer AO and handling Editor declared their shared affiliation.
