# Supplementary material for: Patients with Chronic Obstructive Pulmonary Disease Suffer from Worse Periodontal Health—Evidence from a Meta-Analysis
Source: Front Physiol. 2018 Jan 25;9:33. doi: 10.3389/fphys.2018.00033 (PMC5788941; doi:10.3389/fphys.2018.00033)
Supplement: Supplementary file 1 [file Table1.DOCX]

Supplementary Material

**Patients with Chronic Obstructive Pulmonary Disease Suffer from Worse Periodontal Health—Evidence from a Meta-Analysis**

Quan Shi ^1#^, Bin Zhang ^1#^, Helin Xing ^1^, Shuo Yang ^1^, Juan Xu ^1*^, Hongchen Liu ^1*^

^1^ Institute of Stomatology, Chinese PLA General Hospital, Beijing, China

*** Correspondence:**Hongchen Liu
liuhc301@hotmail.com

Juan Xu
newxj@hotmail.com

^#^ These authors contributed equally to this work.

**Table S1**. Sensitivity analysis performed by sequential removing one study

| **Index** | **Study name** | **WMD** | **95%CI** | **P** |
| --- | --- | --- | --- | --- |
| **PD** | **Total** | **0.261** | **0.020-0.501** | **0.033** |
|  | Terashima T 2016 | 0.196 | -0.052-0.445 | 0.122 |
|  | Bhavsar NV2015 | 0.183 | -0.061-0.427 | 0.142 |
|  | Oztekin G 2014 | 0.317 | 0.065-0.570 | 0.014 |
|  | Peter KP 2013 | 0.231 | -0.037-0.498 | 0.091 |
|  | Ledic K 2013 | 0.239 | -0.017-0.496 | 0.068 |
|  | Yildirim E 2012 | 0.330 | 0.086-0.574 | 0.008 |
|  | Si Y 2012 | 0.290 | 0.002-0.578 | 0.048 |
|  | Wang Z 2009 | 0.321 | 0.079-0.562 | 0.009 |
| **CAL** | **Total** | **0.480** | **0.280-0.681** | **0.000** |
|  | Bhavsar NV 2015 | 0.410 | 0.226-0.594 | 0.000 |
|  | Öztekin G 2014 | 0.511 | 0.311-0.710 | 0.000 |
|  | Peter KP 2013 | 0.447 | 0.222-0.672 | 0.000 |
|  | Ledić K 2013 | 0.434 | 0.239-0.629 | 0.000 |
|  | Vadiraj S 2013 | 0.479 | 0.269-0.688 | 0.000 |
|  | Yıldırım E 2012 | 0.538 | 0.348-0.728 | 0.000 |
|  | Si Y 2012 | 0.456 | 0.245-0.667 | 0.000 |
|  | Deo V 2009 | 0.501 | 0.291-0.712 | 0.000 |
|  | Wang Z 2009 | 0.505 | 0.300-0.710 | 0.000 |
|  | Scannapieco FA 2001 | 0.502 | 0.292-0.712 | 0.000 |
| **PI** | **Total** | **0.226** | **0.043-0.408** | **0.016** |
|  | Bhavsar NV 2015 | 0.139 | -0.041-0.320 | 0.130 |
|  | Oztekin G 2014 | 0.232 | 0.008-0.456 | 0.042 |
|  | Peter KP 2013 | 0.299 | 0.148-0.450 | 0.000 |
|  | Si Y 2012 | 0.227 | -0.058-0.512 | 0.118 |
|  | Wang Z 2009 | 0.232 | 0.004-0.461 | 0.046 |
| **Remaining teeth** | **Total** | **-3.726** | **-5.120- -2.33** | **0.000** |
|  | Terashima T 2016 | -2.942 | -4.299- -1.584 | 0.000 |
|  | Chung JH 2016 | -3.921 | -5.710- -2.133 | 0.000 |
|  | Oztekin G 2014 | -3.696 | -5.236- -2.157 | 0.000 |
|  | Ledic K 2013 | -3.304 | -4.758- -1.849 | 0.000 |
|  | Si Y 2012 | -4.247 | -5.817- -2.677 | 0.000 |
|  | Wang Z 2009 | -4.213 | -5.792- -2.634 | 0.000 |
|  | Hayes C 1998 | -4.239 | -5.812- -2.655 | 0.000 |

WMD: weighted mean difference, CI: confidence intervals, PD: probing depth, CAL: clinical attachment loss, PI: plaque index.
